# Frequency-Dependent Changes in Wavelet-ALFF in Patients With Acute Basal Ganglia Ischemic Stroke: A Resting-State fMRI Study

**DOI:** 10.1155/np/8003718

**Published:** 2025-02-25

**Authors:** Shuolin Liang, Di He, Bin Qin, Chaoguo Meng, Jianxin Zhang, Lanfen Chen, Zhijian Liang

**Affiliations:** ^1^Department of Neurology, The First Affiliated Hospital of Guangxi Medical University, Nanning, Guangxi Zhuang Autonomous Region, China; ^2^School of Information and Electronics Technology, Jiamusi University, Jiamusi, Heilongjiang Province, China; ^3^School of Foreign Studies, China University of Petroleum (East China), Qingdao, Shandong Province, China; ^4^School of Medical Imaging, Shandong Second Medical University, Weifang, Shandong Province, China

**Keywords:** acute basal ganglia ischemic stroke, multifrequency bands, regional neural activity, resting-state functional magnetic resonance imaging, Wavelet-ALFF

## Abstract

**Background and Purpose:** Motor impairment is a common occurrence in patients with acute basal ganglia (BG) ischemic stroke (ABGIS). However, the underlying mechanisms of poststroke motor dysfunction remain incompletely elucidated. In this study, we employed multifrequency band wavelet transform-based amplitude of low-frequency fluctuations (Wavelet-ALFFs) to investigate the alterations of spontaneous regional neural activity in patients with ABGIS.

**Methods:** A total of 39 ABGIS patients with motor dysfunction and 45 healthy controls (HCs) underwent resting-state functional magnetic resonance imaging. Wavelet-ALFF values were calculated in the conventional frequency band (0.01–0.08 Hz), slow-5 frequency band (0.01–0.027 Hz), and slow-4 frequency band (0.027–0.073 Hz). A two-sample *t*-test was performed to compare the Wavelet-ALFF values between the two groups with sex as a covariate and Gaussian random field (GRF) theory (voxel *p* < 0.001, cluster *p* < 0.05, two-tailed) was used for the multiple corrections. Furthermore, spearman correlation analysis was performed to assess the relationship between alterations in regional neural activity between Fugl–Meyer Assessment (FMA) and National Institutes of Health Stroke Scale (NIHSS) scores.

**Results:** In comparison to HCs, patients with ABGIS showed significantly increased Wavelet-ALFF in the left middle temporal gyrus (MTG) and decreased Wavelet-ALFF in the right inferior frontal operculum (IFO) across all three frequency bands (conventional, slow-4, and slow-5). In the left superior occipital gyrus (SOG), Wavelet-ALFF was decreased in the conventional frequency band but increased in the slow-4 frequency band. Additionally, patients with ABGIS demonstrated reduced Wavelet-ALFF in the right superior temporal gyrus (STG) in the conventional and slow-4 frequency bands. In the slow-5 frequency band, increased Wavelet-ALFF was observed in the left calcarine cortex (CC), left middle frontal gyrus (MFG), left supramarginal gyrus (SMG), and left postcentral gyrus (PCG), while decreased Wavelet-ALFF was noted in the right precuneus (PCu). Correlation analysis revealed that increased Wavelet-ALFF in the left CC in the slow-5 frequency band was positively correlated with the FMA score. No other correlations were detected in the conventional and slow-4 frequency bands.

**Conclusions:** The altered spontaneous neural activity was frequency-specific in patients with ABGIS, and the slow-5 frequency band exhibited better results. Furthermore, the relationship between spontaneous brain activity and clinical characteristics highlighted patterns of neural alterations associated with motor dysfunction. These findings may provide novel insights into the neural mechanisms underlying motor dysfunction in ABGIS.

## 1. Introduction

Stroke, a leading cause of death and disability worldwide, is associated with motor deficiency regardless of the lesion site [1]. Approximately 70% of strokes worldwide are ischemic, with a higher proportion in the United States at about 85%–87% [2]. A national survey on stroke cases in China over 20 years reveals that ischemic stroke has the highest prevalence, with 2.87 million cases reported in 2019 alone [3]. Furthermore, it is also found that more than 75% of patients with acute ischemic stroke (AIS) have motor impairments, which places a heavy burden on both patients and their families [4]. However, the mechanisms underlying motor dysfunction after stroke have not been fully understood. Gaining insights into these mechanisms is crucial to the enhancement of therapeutic interventions and rehabilitation strategies for motor function recovery in poststroke. The etiology of neurological dysfunction and reorganization may depend on a number of factors, such as the location of the stroke-induced lesion. The basal ganglia (BG), characterized by a rich vascular network, are more susceptible to stroke [5]. This region is involved in higher-order motor control, including planning and executing complex motor synergies. Additionally, the BG also plays a critical role in motor skill acquisition by coordinating dynamic interactions between multiple neural networks within the frontal and parietal cortex [6]. Consequently, understanding brain plasticity following BG stroke is vital for elucidating motor dysfunction.

In recent decades, the advancement of functional imaging techniques has significantly enhanced our understanding of the neural mechanisms underlying various neuropsychiatric disorders and their rehabilitation. Resting-state functional magnetic resonance imaging (rs-fMRI) has been used as a noninvasive method to study the intrinsic functional activity and connectivity patterns of the brain. Previous studies have found abnormal regional brain activity in patients with stroke disorders, which revealed a secondary functional reorganization of the brain in these patients [7, 8]. In addition, information on brain plasticity after BG ischemic stroke provided valuable insights into the mechanisms of motor dysfunction. In a study using single-photon emission computed tomography, Choi et al. [9] reported that the bilateral primary motor cortex and ipsilateral parietal lobe cerebrovascular reserve were associated with the recovery of upper motor function after BG stroke. Fu et al. [10] conducted a task-based fMRI study and revealed that the cerebellum may play an intermediary role during motor recovery after BG infarction. However, few studies have investigated motor dysfunction in patients with acute BG ischemic stroke (ABGIS) using rs-fMRI.

Previous rs-fMRI studies mainly focused on conventional frequency band, that is, 0.01–0.08 Hz. However, Buzsáki and Draguhn [11] observed that different frequency bands of brain oscillations are associated with different neural functions, so they introduced a classification system for these bands. Specifically, the conventional low-frequency band (0.01–0.08 Hz) was associated with intrinsic neural activity and had physiological significance [12]. The slow-4 band (0.027–0.073 Hz) was found to be particularly effective in capturing signals related to gray matter neural activity, [13] while the slow-5 band (0.01–0.027 Hz) showed sensitivity across a wide range of cerebral cortical areas, effectively reflecting neural activity [14]. Given that different frequency bands respond to different neurological changes, the use of multi-band analyses can provide a more comprehensive understanding of abnormalities in brain function across different frequency bands, thereby enhancing the accuracy and specificity of the test. Recently, our study demonstrated the changes in amplitude of low-frequency fluctuations (ALFFs) and fractional ALFF (fALFF) in three frequency bands in patients with ABGIS compared with healthy controls (HCs), revealing that the abnormal low-frequency oscillation amplitude was related with different frequency bands [15]. Another study using regional homogeneity (ReHo) in multifrequency bands revealed alterations in brain activity in patients with ABGIS, providing a new insight into the pathogenesis of ABGIS [16]. Taken together, previous studies applying three common measures (ALFF, fALFF, and ReHo) suggest that patients with ABGIS exhibit alterations in intrinsic brain activity, which depend on the frequency bands of rs-fMRI data [15, 16].

Conventionally, the fast Fourier transform (FFT), which converts a time series into the frequency domain using a sinusoidal function, was used to calculate ALFF [17]. However, the irregular shape of mother wavelets is more suitable for modeling biological signals than periodic sine function decomposition [17, 18]. Wavelet transform-based ALFF (Wavelet-ALFF) was introduced by Luo et al. [19] for the analysis of rs-fMRI signals, and it showed superior sensitivity and reproducibility than FFT-ALFF. In particular, Wavelet-ALFF employs continuous wavelet transform (CWT) to decompose time series data into both temporal and frequency domains, thereby facilitating a more detailed analysis of brain signals than FFT-ALFF. Unlike FFT, which employs fixed sinusoidal functions, Wavelet-ALFF utilizes mother wavelets with irregular shapes that can adaptively adjust to the localized and transient nature of fMRI signals. This adaptability allows Wavelet-ALFF to capture subtle variations in brain activity by analyzing the data at multiple scales, thus providing a detailed representation of both high- and low-frequency fluctuations. Consequently, Wavelet-ALFF exhibits enhanced sensitivity and reproducibility in effectively detecting and quantifying spontaneous brain activity with greater precision, making it particularly suited for analyzing complex and nonstationary biological signals [19]. Additionally, a recent multicenter study with eyes-closed and eyes-open datasets indicated that Wavelet-ALFF had the best reliability and validity, suggesting that Wavelet-ALFF might be a powerful metric for inspecting spontaneous regional brain activities [17]. However, there appear to be few studies using Wavelet-ALFF to explore the spontaneous changes in brain activity among patients with ABGIS.

In this study, Wavelet-ALFF was applied to investigate alterations in spontaneous regional brain activity in conventional (0.01–0.08 Hz), slow-5 (0.01–0.027 Hz), and slow-4 (0.027–0.073 Hz) frequency bands. Furthermore, correlations between the altered brain regions in patients with ABGIS and clinical features were also investigated, and the results may strengthen our understanding of the neural mechanisms underlying motor dysfunction in ABGIS.

## 2. Material and Methods

### 2.1. Participants

Thirty-nine patients with ABGIS and 45 age-matched HCs (mean age, 55.82 ± 10.68 and 55.11 ± 11.46 years, respectively) were recruited from 2019 to 2021. All patients with ABGIS were recruited from the Department of Neurology of the First Affiliated Hospital of Guangxi Medical University. HCs were recruited from the local community through advertisements. The eligibility criteria for patients were (1) age between 30 and 75 years, (2) full admission history (within 10 days after symptom onset), clinical diagnosis of first-onset ABGIS with motor dysfunction and National Institutes of Health Stroke Scale (NIHSS) scores from 0 to 16, [15] (3) unilateral infarction involving the BG, (4) absence of other brain lesions or prior infarcts, (4) no contraindication to MRI, (5) right-handedness before stroke, (6) sufficient cognitive abilities. The exclusion criteria for patients were (1) inability to perform clinical examinations, owing to severe aphasia, auditory, and/or visual disorders; (2) presence or a history of psychiatric or other neurological diseases; (3) a history of substance or alcohol abuse; and (4) excessive head motion during rs-fMRI scanning (the cardinal directions [*x*, *y*, *z*] more than 3.0 mm and a maximum spin [*x*, *y*, *z*] greater than 3°). The eligibility criterion for HCs included no history of neurological and/or psychiatric conditions. This study was approved by the Ethics Committee of the First Affiliated Hospital of Guangxi Medical University. Written informed consent was obtained from all participants.

### 2.2. Neurological Assessments

Motor function, balance, sensation, and joint function of the upper- and lower-limb motor scores were evaluated using the Fugl–Meyer Assessment (FMA) [20]. The scale ranges from 0 to 100 points, with upper- and lower-limb motor scores accounting for 66 and 34 points, respectively. Higher scores indicate better motor function. The NIHSS quantifies the severity of neurological deficits in patients with stroke, with a score range of 0–42, graded: mild ≤7, moderate 8–16, and severe ≥17 [21]. All neurological assessments were conducted independently by two neurologists.

### 2.3. MRI Data Acquisition

All MRI data were acquired at the Department of Radiology, First Affiliated Hospital of Guangxi Medical University, using a Siemens MAGNETOM Prisma 3.0-T MRI scanner. Participants were instructed to remain awake, relaxed, and motionless as much as possible while undergoing the fMRI scan. The 64-pass phased-array head coil was adopted, and the standard scanning protocol was strictly followed.

Echoplanar imaging was used to acquire rs-fMRI with the following parameters: repetition time (TR) = 2000 ms, echo time (TE) = 35 ms, flip angle (FA), 90°; field of view (FOV), 240 × 240 mm^2^; voxel size = 2.6 × 2.6 × 3 mm^3^; matrix, 64 × 64; gap, 0 mm; slice number, 40. The session lasted for 6 min and 12 s.

A magnetization-prepared rapid gradient echo was used to record anatomical 3D-MPRAG T1-weighted images: TR, 2300 ms; TE, 2:98 ms, reverse time, 900 ms; FOV, 256 × 256 mm^2^; voxel size, 1 × 1 × 1 mm^3^; matrix, 256 × 256; gap, 0 mm; and slice number, 176. The session duration was 5 min and 21 s.

### 2.4. Data Preprocessing

The rs-fMRI data preprocessing and statistical analysis were performed using RESTplus V1.24 (http://www.restfmri.net) and SPM12 (http://www.fil.ion.ucl.ac.uk/spm/software/spm12/) based on MATLAB R2018a (http://uk. mathwork. com products/Matlab). Multiple comparison corrections were performed using Data Processing & Analysis for Brain Imaging (DPABI) V6.0 (http://rfmri.org/dpabi). The preprocessing stage consisted of the following steps: (1) removal of the first 10 time points to help the longitudinal magnetization reach a steady state; (2) slice timing correction; (3) realignment; (4) normalization: standard Montreal Neurological Institute (MNI) spatial normalization was performed on the realigned images, and the voxels were resampled to 3 × 3 × 3 mm^3^; (5) spatial smoothing with full width at half maximum kernel size of 4 mm; (6) nuisance covariate regression: Nuisance regressors included Friston-24 motion parameters, [22] white matter and cerebrospinal fluid signals; (7) removed linear trends; (8) filters: temporal band-pass filtering was performed in three frequency bands: conventional (0.01–0.08 Hz), slow-5 (0.01–0.027 Hz), and slow-4 (0.027–0.073 Hz).

### 2.5. Wavelet-ALFF Calculation

In this study, we calculated the Wavelet-ALFF values in three frequency bands (conventional, slow-5 and slow-4). Wavelet-ALFF analysis was performed using RESTplus V1.24, with calculations based on CWT. Luo et al. [19] calculated Wavelet-ALFF values by adding the coefficients at all time points for each frequency point and then averaging the coefficients across a given band of frequencies. The Wavelet-ALFF values of each voxel were then divided by the global mean of the Wavelet-ALFF values for standardization. Notably, after the Wavelet-ALFF calculations, the maps of 18 patients with right-sided lesions were flipped to the left using RESTplus V1.27 to remove the possible effects of lateralization [23, 24].

### 2.6. Statistical Analysis

Statistical analyses were performed using Statistical Product and Service Solutions 26.0 (SPSS 26.0; IBM, Armonk, NY, United States). Categorical variables were presented as *n*, whereas continuous variables were presented as the mean ± standard deviation. Sex differences, hypertension, type 2 diabetes, hyperlipidemia, current smoking, and alcohol consumption between patients with ABGIS and HCs were compared using the chi-square test. A two-sample *t*-test was performed to compare age and education differences between the two groups. All tests performed on demographic data were two-tailed, and a *p*-value < 0.05 was considered significant.

Two-sample *t*-tests were performed to compare the differences of Wavelet-ALFF between patients with ABGIS and HCs with sex as a covariate in each frequency band. To eliminate the influence of head motion, Frame-wise displacement (FD, Jenkinson) parameters were regressed in the two-sample *t*-test [25]. The resultant *t*-maps were corrected for multiple comparisons based on Gaussian random field (GRF) theory, with a two-tailed voxel-level threshold of *p* < 0.001 and a two-tailed cluster-level threshold of *p* < 0.05 [26, 27].

Spearman correlation analyses were performed to assess the correlation between Wavelet-ALFF values and FMA scores, as well as NIHSS scores. Correlations were considered significant at a threshold of *p* < 0.05.

## 3. Results

### 3.1. Demographic and Clinical Characteristics of the Participants

A total of 39 patients with ABGIS (30 men; mean age: 55.82 ± 10.68 years) and 45 HCs (20 men; mean age: 55.11 ± 11.46 years) were enrolled in this study, of which 18 patients had lesions on the right side and 21 patients had lesions on the left side. The demographic and clinical data of all participants are summarized in Table 1. There were no significant differences between the two groups in terms of age (*p*=0.771) and education (*p*=0.573). However, there were significant sex differences between the two groups *i* (*p*=0.002). Moreover, there were also significant differences between the two groups in the following aspects: hypertension (*p* < 0.001), type 2 diabetes (*p*=0.021), hyperlipidemia (*p*=0.003), current smoking (*p* < 0.001), and alcohol consumption (*p*=0.006).

### 3.2. Wavelet-ALFF Analysis in Multifrequency Bands

**Figure 1:**
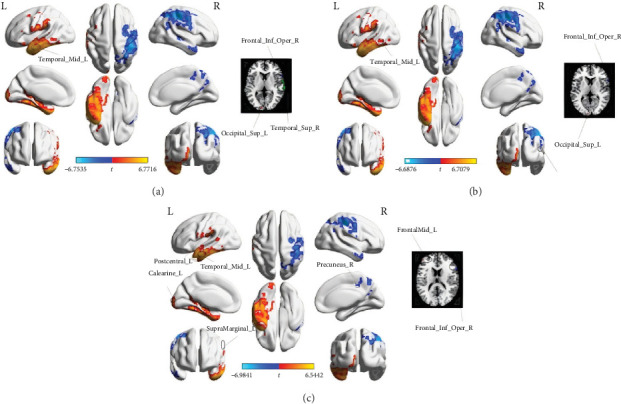
Brain regions with significant differences in Wavelet-ALFF in three different frequency bands between ABGIS and HCs groups. (A) Brain regions with differences in Wavelet-ALFF in conventional frequency band. (B) Brain regions with differences in Wavelet-ALFF in slow-4 frequency band. (C) Brain regions with differences in Wavelet-ALFF in slow-5 frequency band. ABGIS, basal ganglia ischemic stroke; Calcarine_L, left calcarine cortex; Frontal_Inf_Oper_R, right inferior frontal operculum; Frontal_Mid_L, left middle frontal gyrus; HCs, healthy controls; Occipital_Sup_L, left superior occipital gyrus; Postcentral_L, left postcentral gyrus; Precuneus_R, right precuneus; SupraMarginal_L, left supramarginal gyrus; Temporal_Mid_L, left middle temporal gyrus; Temporal_Sup_R, right superior temporal gyrus; Wavelet-ALFF, wavelet transform-based amplitude of low-frequency fluctuation.

In the conventional frequency band, patients with ABGIS displayed significantly increased Wavelet-ALFF values in the left middle temporal gyrus (MTG) and decreased Wavelet-ALFF values in the right inferior frontal operculum (IFO), left superior occipital gyrus (SOG) and the right superior temporal gyrus (STG) compared with HCs (Table 2 and Figure 1).

Similarly, in the slow-4 frequency band, patients with ABGIS displayed significantly increased Wavelet-ALFF values in the left MTG and the left SOG while significantly decreased Wavelet-ALFF values in the right IFO, and right STG (Table 2 and Figure 1).

In the slow-5 frequency band, patients with ABGIS displayed significantly increased Wavelet-ALFF values in the left MTG, left calcarine cortex (CC), left middle frontal gyrus (MFG), left postcentral gyrus (PCG), and left supramarginal gyrus (SMG). However, significantly decreased Wavelet-ALFF values were observed in the IFO and the right precuneus (PCu) in patients with ABGIS compared with HCs (Table 2 and Figure 1).

### 3.3. Correlations Between Wavelet-ALFF Values and FMA Scores

The correlations between Wavelet-ALFF values of these abnormal brain regions and FMA scores were also explored. In the conventional frequency band, changes in Wavelet-ALFF values were not significantly correlated with FMA scores (e.g., MTG: *r* = 0.175, *p*=0.294; IFO: *r* = −0.042, *p*=0.805; SOG: *r* = 0.241, *p*=0.145; STG: *r* = −0.259, *p*=0.117) (Figure S1. A1–4). Similarly, no significant correlation was observed in the slow-4 band (MTG: *r* = 0.181, *p*=0.278; IFO: *r* = -0.051, *p*=0.763; SOG: *r* = 0.220, *p*=0.184; STG: *r* = −0.260, *p*=0.115) (Figure S1. B1–4). In the slow-5 frequency band, the Wavelet-ALFF value of the left CC was significantly and positively correlated with the FMA score (*r* = 0.378, *p*=0.019). However, there was no significant correlation between other brain regions, including the left PCG (*r* = 0.233, *p*=0.159), right PCu (*r* = 0.069, *p*=0.679), left SMG (*r* = 0.101, *p*=0.548), left IFO (*r* = 0.009, *p*=0.956), left MTG (*r* = 0.142, *p*=0.395) and the left MFG (*r* = 0.044, *p*=0.794), and FMA scores in the slow-5 frequency band (Figure S1.C1–7).

### 3.4. Correlations Between Wavelet-ALFF Values and NIHSS Scores

Correlation analyses showed that there were no significant correlations between Wavelet-ALFF values of the abnormal brain regions and NIHSS scores. Specifically, in the conventional frequency band, there was no significant correlation between NIHSS score and Wavelet-ALFF values of the altered brain regions, including the left MTG (*r* = −0.086, *p*=0.607), right IFO (*r* = 0.093, *p*=0.579), left SOG (*r* = −0.280, *p*=0.089), or right STG (*r* = −0.089, *p*=0.595) (Figure S1. A5–8). Similarly, in the slow-4 frequency band, no significant correlations were found between the NIHSS scores and the left MTG (*r* = −0.089, *p*=0.595), right IFO (*r* = 0.103, *p*=0.540), left SOG (*r* = −0.256, *p*=0.121), as well as right STG (*r* = −0.256, *p*=0.121) (Figure S1. B5–8). In the slow-5 frequency band, there were also no significant correlations between NIHSS scores and the Wavelet-ALFF values of the following regions: left CC (*r* = −0.278, *p*=0.091), left PCG (*r* = −0.210, *p*=0.205), left MFG (*r* = 0.147, *p*=0.380), left SMG (*r* = −0.155, *p*=0.352), left MTG (*r* = −0.076, *p*=0.650), left IFO (*r* = 0.038, *p*=0.821), and right PCu (*r* = −0.103, *p*=0.538) (Figure S1. C8–14).

## 4. Discussion

In this study, we employed the Wavelet-ALFF approach to evaluate alterations in brain activity across distinct frequency bands in patients with ABGIS. The results demonstrated frequency-specific alterations in spontaneous neural activity in various brain regions among ABGIS patients, particularly in the conventional, slow-4, and slow-5 bands. Specifically, in the conventional band, patients with ABGIS showed increased Wavelet-ALFF values in the left MTG and decreased values in the right IFO, left SOG, and right STG. In the slow-4 band, significant increases were observed in the left MTG and left SOG, while decreases were found in the right IFO and right STG. Similarly, in the slow-5 band, patients exhibited increased Wavelet-ALFF values in the left MTG, CC, MFG, PCG, and SMG, along with decreased values in the IFO and right PCu. These findings suggest that neural activity changes in ABGIS patients are closely associated with specific frequency bands, providing new insights into the underlying neural mechanisms of ABGIS.

Consistent alterations in the MTG and IFO across all three frequency bands suggest that these two regions may play a critical role in modulating neural activity in ABGIS patients. The MTG, located in the temporal lobe, is involved in various functions such as language, memory, and visual information processing and plays a crucial role in developing visual-motor-based speech networks [28]. The MTG participates in the integration of multifaceted vision-motion operations, such as coordinating motion processes through visual signals, which holds particular importance in motion planning and fine motion control [29]. Additionally, it plays a significant role in regulating motor feedback monitoring within neural networks [30]. In patients who suffered a stroke, motor dysfunction may trigger a compensatory mechanism involving visual function, leading to increased activation of the MTG [29]. In the present study, increased Wavelet-ALFF in the MTG of patients with ABGIS may reflect this process, suggesting that this region plays an important role in the recovery of motor control in patients with ABGIS. The IFO is a component of the prefrontal cortex and is highly associated with language, cognitive, and emotional functions [31, 32]. Pineda-Pardo et al. [33] observed that the application of transcranial static magnetic field stimulation (tSMS) in the supplementary motor area (SMA) among healthy subjects increased SMA brain activity and enhanced functional connectivity (FC) between the paracentral lobule and the IFO. This was associated with a reduction in the time required for motor initiation at the behavioral level, along with decreased reaction time for motor selection. It is proposed that the neural loop connecting the inferior frontal gyrus and the SMA plays a role in regulating motor function, particularly in maintaining a balance between motor speed and accuracy. Moreover, the IFO is believed to be involved in generating complex hand gestures. Theta burst stimulation of the IFO has been observed to disrupt the generation of such gestures [34]. In patients with ABGIS, lesions in the BG region may impair the connectivity between the IFO and SMA, contributing to motor dysfunction [35]. This also explains why the Wavelet-ALFF values for IFO were reduced in the present study.

In both conventional and slow-4 frequency bands, the left SOG and right STG were both observed alterations. The SOG, situated in the occipital lobe, is primarily involved in visual information processing. It is also closely associated with the temporal evolution of planning and executing movement directions, especially the visual-motor transition [36]. Vaina et al. [37] found that SOG played a similarly crucial role in the visual-motor transformation process in stroke patients, potentially compensating for other damaged components of the visual-motor network. This may be associated with the brain's capacity to initiate a certain degree of compensatory visual-motor coordination during poststroke motor dysfunction. Such compensatory mechanisms may facilitate partial recovery of motor function during visual-motor transitions. In addition, the STG, which is located in the temporal lobe and mainly involved in speech, hearing, cognition, and executive attention, [38] also displayed alterations. The STG is also closely related to spatial awareness, proprioception, and postural perception. Rousseaux et al. [39] demonstrated that the STG significantly contributes to the regulation and maintenance of body posture. After the STG injury, the patient's proprioceptive ability was weakened, leading to a midline bias of the body towards the side of the lesion. In this study, although there was no structural damage in the STG among ABGIS patients, there was a change in the Wavelet-ALFF in this region, suggesting that the STG may affect the patients' motor function through alternative pathways. Cortico-BG circuits are key to the acquisition and execution of motor skills, coordinating and controlling fine motor movements through indicating neural connections. During the process of motor learning, neural activity in the cortex and BG undergoes adaption and remodeling, resulting in more structured and relevant neural movement patterns that align with the learned movement throughout continuous movement [40–42]. In stroke patients, the STG is not directly involved in the onset of motor dysfunction. However, given the close link between the STG and proprioception, the proprioceptive impairments observed in poststroke may result from either a disruption in the ability to sense limb position or a failure to effectively process proprioceptive information needed to guide subsequent movements. Thus, even if the STG itself is not damaged, a lesion in the BG may lead to functional impairment of the STG, specifically an inability to facilitate information interactions with the cortex via the BG—resulting in proprioceptive information that is not properly conveyed to the STG and consequently diminishing neural activity in the region [43–45].

The present study further identified altered neural activity in additional brain regions within the slow-5 frequency band. Specifically, the results showed increased activity in the CC, PCu, MFG, PCG, and SMG, which are associated with visual function, cognitive function, and sensory-motor processing, respectively. A previous study conducted by Li et al. [46] revealed a significant reduction in FC between the left CC and the contralateral motor cortical layer in ABGIS patients. This finding indicates that neural activity alterations in poststroke are not confined to the motor cortex but also extend to the visual cortex [47]. The CC, serving as the primary visual cortex, is responsible for processing visual signals, which is vital for the motor function [48]. This provides a potential explanation for the increase in neural activity in the CC, which may be a compensatory mechanism to maintain visual-motor integration after a reduction in FC between the CC and the motor cortex. The PCu is part of the parietal lobule and is located between the sensorimotor cortices of the paracentral lobule and the parieto-occipital cortex in the central lobule. It plays an important role in cognition, sensory integration, and higher sensory processing [49, 50]. Although the PCu is not directly responsible for movement, it is connected with the prefrontal cortex and the anterior and middle cingulate gyrus, which, in conjunction with the pathways between the cingulate gyrus and the PCu, assist the brain in responding to stimuli and linking to motor areas, such as the SMA via the superior longitudinal fasciculus (SLF), ultimately guiding motor actions. This suggests that the PCu is involved in both cognitive processes and the regulation of bodily movements through connections with other brain regions [51]. In this study, patients with ABIGS showed decreased Wavelet-ALFF values in the PCu, indicating a reduction in neural activity within this region. The PCu processes information from visual, autosensory, and multisensory inputs and allows the integration of sensory and motor information to be involved in higher cognitive functions related to the formation of motor intentions or early motor plans. This indicates that the PCu may, in conjunction with other motor-related regions such as the SMA, operate within a visual-cognitive-motor regulatory network. The SMA, which is implicated in motor planning and execution, plays a pivotal role in coordinating intricate movements, including those necessitating sequential actions or bimanual coordination, which is disrupted in ABIGS [52]. In accordance with the previous study, our results demonstrated that stroke patients exhibited increased activation in the MFG [53]. The MFG is primarily involved in attention, working memory, language-related processing as well as motor planning and decision-making processes due to its extensive white matter connectivity. It integrates sensory information and coordinates with other motor regions to formulate movement strategies. Damage to the MFG can lead to difficulties in initiating and planning voluntary movements, highlighting its role in motor function [54, 55]. The PCG and SMG are involved in the processing of sensory information, motor function, and advanced cognitive tasks. Zhao et al. observed an increased FC between the contralateral SMA and the affected postcentral gyrus in stroke patients when compared to HCs. This indicates that motor recovery may be related to the reorganization of the contralateral motor network, including the enhancement of FC in the PCG [56]. A previous study has shown that PCG and SMG play an important role in the recovery of motor function after stroke. A significant correlation between the structural integrity of white matter (FA values) in PCG-SMG and patients' motor performance suggests that the connection between PCG and SMG is crucial for maintaining normal motor performance and recovery of motor function after stroke [57]. The present study revealed a significant increase in Wavelet-ALFF values in the PCG and SMG, indicating elevated neural activity in these regions. This suggests that these areas are not only involved in sensory processing but also play a key role in motor control and coordination in patients with ABGIS.

Meanwhile, we also conducted a correlation analysis to explore the relationship between altered brain regions and clinical indicators. A positive correlation was observed between Wavelet-ALFF values in the CC and FMA scores in the slow-5 band, suggesting that visual-motor integration may play a key compensatory role in motor function in stroke patients [46, 47]. Nevertheless, no other brain regions demonstrated significant correlations between Wavelet-ALFF values and FMA scores across different frequency bands. This may be attributed to the relatively subtle differences in motor impairment among patients, which may not be sufficient to reveal more widespread correlations in these brain regions. What's more, these altered brain regions are not solely linked to motor control but also to cognitive and emotional processes. For instance, the MFG and IFO, while involved in motor control, are also integral to cognition and emotion. Since the FMA predominantly assesses motor function, it may not fully capture the range of neural activity reflected in Wavelet-ALFF, which likely explains the absence of further significant correlations. Besides, the correlation analysis was also conducted between altered brain regions and NIHSS scores. However, no significant correlations were identified between Wavelet-ALFF values and NIHSS scores. This maybe due to the small sample size, which limiting the detection of correlations. Moreover, the NIHSS scale assesses a comprehensive range of neurological domains, including language, sensory function, motor abilities, and consciousness, which may have hindered the ability to discern specific associations between NIHSS scores and regional brain activity as measured by Wavelet-ALFF.

Though the findings may contribute to understanding the neural mechanisms of stroke, there still exist some limitations. First, the sample size was relatively small, owing to the strict inclusion and exclusion criteria. Second, the infarction lesions of the patients were not all on the same side, and although we performed a lateralization process, we cannot absolutely exclude the influence of lateralization. Finally, we only compared the cross-sectional changes in Wavelet-ALFF in patients with ABGIS and HCs. Subsequent studies will expand the sample size and exclude the effect of lateralization. Furthermore, the temporal longitudinal study of Wavelet-ALFF will continue in order to identify neural activities that depend on temporal changes.

## 5. Conclusion

The present study identified alterations in Wavelet-ALFF across multiple brain regions in patients with ABGIS, with these alterations demonstrating frequency-specific characteristics. Notably, the slow-5 frequency band was more effective than the conventional and slow-4 frequency bands in capturing Wavelet-ALFF alterations, highlighting the frequency-specific sensitivity of the slow-5 band in detecting neural activity changes in ABGIS. Our findings offer novel insights into the neural mechanisms underlying motor dysfunction in ABGIS, suggesting that targeted frequency-specific analyses could enhance future therapeutic strategies.

## Figures and Tables

**Table 1:** Demographic and clinical data of participants.

Characteristics	ABGIS group	HCs	*p*-Value
Number	39	45	—
Age (years)	55.82 ± 10.68	55.11 ± 11.46	0.771
Sex (males/females)	30/9	20/25	0.002^*∗*^
Education (years)	11.67 ± 3.56	12.09 ± 3.28	0.573
Hypertension, no. (%)	28 (71.8)	7 (15.6%)	0.001^*∗*^
Type 2 diabetes, no. (%)	11 (28.2)	4 (8.9)	0.021^*∗*^
Hyperlipidemia, no. (%)	15 (38.5)	5 (11.1)	0.003^*∗*^
Current smoking, no. (%)	22 (68.8)	10 (31.3)	<0.001^*∗*^
Alcohol consumption, no. (%)	22 (56.4)	12 (26.7)	0.006^*∗*^
Left hemispheric lesion, no	21	—	—
Right hemispheric lesion, no	18	—	—
NIHSS score	3.74 ± 2.35	—	—
FMA score	75.15 ± 18.88	—	—
FMA-up	48.62 ± 14.28	—	—
FMA-low	26.54 ± 5.11	—	—

Abbreviations: ABGIS, basal ganglia ischemic stroke; FMA, Fugl–Meyer Assessment; FMA-low, Fugl–Meyer Assessment scale of the lower limbs; FMA-up, Fugl–Meyer Assessment scale of the upper limbs; HCs, healthy controls; NIHSS, National Institutes of Health Stroke Scale.

*⁣* ^*∗*^ *p* < 0.05.

**Table 2:** Brain regions with significant differences in Wavelet-ALFF in three different frequency bands between ABGIS and HCs groups.

Brain regions (AAL)	Number of voxels	MNI coordinate			Peak *t* value
		*x*	*y*	*z*	
Conventional frequency band (0.01–0.08 Hz)					
Temporal_Mid_L	2553	−66	−18	−12	6.7716
Frontal_Inf_Oper_R	2537	63	18	15	−6.7535
Occipital_Sup_L	49	−9	−96	6	−4.8064
Temporal_Sup_R	42	45	−27	−3	−4.1969
Slow-4 frequency band (0.027–0.073 Hz)					
Temporal_Mid_L	2602	−66	−9	15	6.7079
Frontal_Inf_Oper_R	2720	63	18	15	−6.6876
Occipital_Sup_L	41	−9	−96	6	4.7619
Temporal_Sup_R	43	45	−27	−3	−4.1256
Slow-5 frequency band (0.01–0.027 Hz)					
Temporal_Mid_L	1522	−66	−21	−12	6.5442
Frontal_Inf_Oper_R	1625	63	18	18	−6.9841
Calcarine_L	33	−6	−96	6	4.5404
Frontal_Mid_L	180	−57	−6	45	5.5051
Postcentral_L	42	−66	−9	15	5.0506
SupraMarginal_L	101	−54	−30	30	4.8881
Precuneus_R	45	21	−57	21	−4.7745

*Note:* The statistical threshold was set at voxel with *p* < 0.001 and cluster with *p* < 0.05 for multiple comparisons using Gaussian random field (GRF) theory corrected. In every cluster, the first label represents the brain region that peak voxel is located, and the second label is the subregion within the same cluster.

Abbreviations: AAL, automated anatomical labeling; ABGIS, basal ganglia ischemic stroke; Calcarine_L, left calcarine cortex; Frontal_Inf_Oper_R, right inferior frontal operculum; Frontal_Mid_L, left middle frontal gyrus; HCs, healthy controls; Occipital_Sup_L, left superior occipital gyrus; Postcentral_L, left postcentral gyrus; Precuneus_R, right precuneus; SupraMarginal_L, left supramarginal gyrus; Temporal_Mid_L, left middle temporal gyrus; Temporal_Sup_R, right superior temporal gyrus; Wavelet-ALFF, wavelet transform-based amplitude of low-frequency fluctuation.

## Data Availability

The raw data supporting the conclusions of this study will be made available by the corresponding author upon reasonable request, subject to compliance with ethical and legal obligations related to patient confidentiality and institutional data governance policies.

## Supporting Information

Supporting Information Supporting information includes figures illustrating the correlation between FMA and NIHSS scores and different frequency bands (Conventional, Slow-4, and Slow-5).
