# Supplementary material for: Frequency-Dependent Changes in Wavelet-ALFF in Patients With Acute Basal Ganglia Ischemic Stroke: A Resting-State fMRI Study
Source: Neural Plast. 2025 Feb 25;2025:8003718. doi: 10.1155/np/8003718 (PMC11879565; doi:10.1155/np/8003718)
Supplement: Supporting Information — includes figures illustrating the correlation between FMA and NIHSS scores and different frequency bands (Conventional, Slow-4, and Slow-5). [file 8003718.f1.pdf]

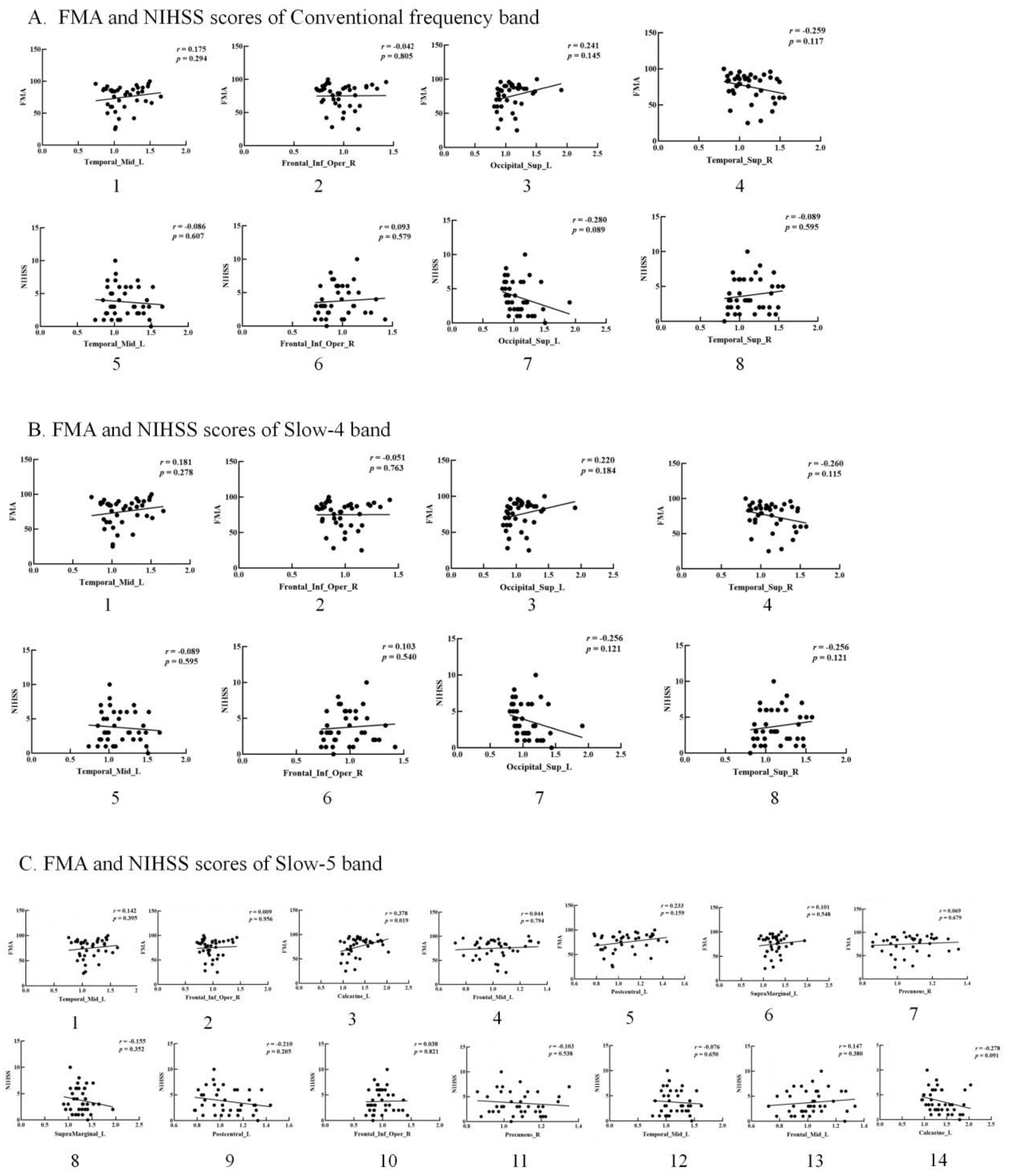

Figure S1 Brain regions showing correlations between FMA/NIHSS scores and Wavelet-ALFF across different frequency bands. (A) Correlation between FMA/NIHSS scores and Wavelet-ALFF in the conventional frequency band. (B) Correlation between FMA/NIHSS scores and Wavelet-ALFF in the slow-4 frequency band. (C) Correlation between FMA/NIHSS scores and Wavelet-ALFF in the slow-5 frequency band.

Temporal_Mid_L, left middle temporal gyrus; Frontal_Inf_Oper_R, right frontal inferior operculum; Occipital_Sup_L, left superior occipital gyrus; Temporal_Sup_R, right superior temporal gyrus; Calcarine_L, left calcarine cortex; Frontal_Mid_L, left middle frontal gyrus; Postcentral_L, left postcentral gyrus; SupraMarginal_L, left supramarginal gyrus; Precuneus_R, right precuneus;
